# Contrasting Relationships Between Anxiety and Intolerance of Uncertainty in Cornelia de Lange and Fragile X Syndromes

**DOI:** 10.1111/jir.70024

**Published:** 2025-08-29

**Authors:** Kayla Smith, Victoria Perry, Laura Groves, Joanna Moss, Chris Oliver, Eve Knight, Tom Patterson, Jacqui Rodgers, Jane Waite, Hayley Crawford

**Affiliations:** ^1^ Mental Health and Wellbeing Unit, Division of Health Sciences, Warwick Medical School University of Warwick Coventry UK; ^2^ Cerebra Network for Neurodevelopmental Disorders University of Birmingham Birmingham UK; ^3^ School of Psychological, Social and Behavioural Sciences Coventry University Coventry UK; ^4^ Birmingham Community Healthcare NHS Foundation Trust Birmingham UK; ^5^ Department of Neuroscience, Psychology and Behaviour University of Leicester Leicester UK; ^6^ School of Psychology University of Birmingham Birmingham UK; ^7^ School of Psychology University of Surrey Guildford UK; ^8^ Population Health Sciences Institute, Sir James Spence Institute Newcastle University, Royal Victoria Infirmary Newcastle Upon Tyne UK; ^9^ School Psychology, College of Health and Life Sciences Aston University Birmingham UK

**Keywords:** anxiety, autism, Cornelia de Lange syndrome, fragile X syndrome, intellectual disability, intolerance of uncertainty

## Abstract

**Background:**

Cornelia de Lange syndrome (CdLS) and fragile X syndrome (FXS) are associated with co‐occurring autism and anxiety. In autistic people, intolerance of uncertainty (IU) mediates the relationship between autistic characteristics and anxiety, but it is not known whether this relationship is evident in these genetic syndromes. Understanding the relationship between autism, anxiety and IU is essential to informing the theoretical frameworks of anxiety in rare genetic syndromes and improving clinical interventions.

**Method:**

Sixty participants with CdLS or FXS participated in a questionnaire‐based study to examine the association between autistic characteristics, anxiety and IU.

**Results:**

IU mediated the association between autism and anxiety in participants with CdLS but not in participants with FXS.

**Conclusions:**

These results suggest that other factors may contribute to the autism‐anxiety relationship in FXS, and highlight the merit of syndrome‐specific approaches to the study of anxiety. Recommendations are made for intervention‐based research to ameliorate anxiety in CdLS.

Rare genetic syndromes associated with intellectual disability (ID) are often characterised by an increased prevalence of co‐occurring conditions such as autism (Bush and Scott 2021; Richards et al. 2015) and anxiety (Basile et al. 2007; Cordeiro et al. 2011; Dykens et al. 2000) with notable heterogeneity in the presentation of each condition across syndromes (Bozhilova et al. 2023; Crawford et al. 2017; Groves et al. 2022; Moss and Howlin 2009). Anxiety is also common in autistic people, with an estimated prevalence rate of 40% (Hollocks et al. 2019; Strang et al. 2012; van Steensel et al. 2011; van Steensel and Heeman 2017), highlighting the need to understand the relationship between autism and anxiety.

Cornelia de Lange syndrome (CdLS) and fragile X syndrome (FXS) are rare genetic syndromes associated with mild to profound ID along with co‐occurring autism and anxiety. CdLS affects approximately 1 in 10 000 to 30 000 newborns (Kline et al. 2007; Kline et al. 2018; Mannini et al. 2013) and results from mutations in the *NIPBL* (Krantz et al. 2004), *SMC1A*, *HDAC8*, *RAD21* (Deardorff et al. 2012; Deardorff et al. 2016), *BRD4*, *ANKRD11* (Kline et al. 2018) and *SMC3* (Revenkova et al. 2009) genes as well as mosaicism for *NIPBL* mutations (Ansari et al. 2014; Huisman et al. 2013), which disrupt gene regulation during early critical development. The CdLS behavioural phenotype includes self‐injurious behaviour, aggression, hyperactivity, deficits in expressive language relative to receptive language and overall adaptive behaviour, and autism‐like characteristics (e.g., repetitive behaviours, decreased sociability and communication difficulties; Ajmone et al. 2014; Basile et al. 2007; Berney et al. 1999; Kline et al. 2018; Mulder et al. 2017; Oliver et al. 2008; Parisi et al. 2015; Srivastava et al. 2021).

FXS affects approximately 1 in 7000 males and 1 in 11 000 females (Hunter et al. 2014) and is the most common cause of inherited ID as well as the leading known genetic cause of autism. FXS results from an expansion mutation of the CGG trinucleotide repeat of the fragile X messenger ribonucleoprotein 1 (*FMR1*) gene on the X chromosome. Consequently, females are generally differentially affected due to the protective effect of a second unaffected X chromosome (Clifford et al. 2007). The FXS behavioural phenotype includes social avoidance *and* social interest, language and motor deficits, hyperactivity and impulsivity, physiological hyperarousal, and autism‐like characteristics (e.g., sensorimotor‐based repetitive behaviours and gaze aversion; Chromik et al. 2019; Ezell et al. 2021; Garber et al. 2008; Hogan et al. 2021; Klusek et al. 2015; Marlborough et al. 2021; McDuffie et al. 2015; Roberts, Crawford, Hogan, et al. 2019; Roberts et al. 2016).

The association between autistic characteristics^1^ and the behavioural phenotype of CdLS and FXS is well supported by previous research. Approximately 43% of people with CdLS and 30% of males with FXS (Richards et al. 2015) meet the diagnostic criteria for a separate diagnosis of co‐occurring autism. However, there are subtle differences in the presentation and severity of autistic characteristics in people with CdLS and FXS compared with those with nonsyndromic autism (Bozhilova et al. 2023). For example, people with CdLS show less frequent autism‐related repetitive behaviours and stereotyped speech than autistic people but similar levels of difference in social interaction and communication (Moss, Howlin, et al. 2013; Moss et al. 2012). Additionally, boys with FXS are less likely to display differences in social reciprocity (e.g., social smiling) compared with autistic boys of the same age (McDuffie et al. 2015). These results suggest that, despite similarities in characteristics, nuanced differences in autism presentation may make it challenging to differentiate autism from the inherent behavioural phenotype of rare genetic syndromes, yet it also affords the opportunity to examine the relationship between profiles of autistic characteristics and anxiety.

Defining co‐occurring autism within the context of the behavioural phenotype of CdLS and FXS is further complicated given the high prevalence of co‐occurring anxiety in these syndromes. Up to 64% of people with CdLS (Basile et al. 2007; Berney et al. 1999; Gualtieri 1991; Kline et al. 2007) and approximately 42% of males with FXS (Edwards et al. 2022) experience clinically significant anxiety. Common anxiety disorders in CdLS include social anxiety, separation anxiety, generalised anxiety and specific phobias (Crawford et al. 2020; Crawford et al. 2017; Groves et al. 2022; Kline et al. 2007; Richards et al. 2009) compared with specific phobia and social anxiety in FXS (Cordeiro et al. 2011; Ezell et al. 2019; Groves et al. 2022). However, similar to the profile of autism, there is significant variability in severity and breadth of anxiety symptomatology in people with CdLS and FXS (Crawford et al. 2017; Groves et al. 2022). Furthermore, behavioural indicators of anxiety may overlap with behavioural indicators of autism, complicating differentiation. For example, social difficulties are associated with anxiety (Crawford et al. 2020; Moss et al. 2008; Nelson et al. 2017; Richards et al. 2009; Thurman et al. 2014) and autism (Ellis et al. 2020; Moss, Howlin, et al. 2013; Moss, Oliver, et al. 2013; Roberts, Crawford, Will, et al. 2019; Roberts et al. 2007) in people with CdLS and FXS. Despite the degree of overlap in behavioural indicators, anxiety is considered a discrete diagnosis rather than a diagnostic feature of autism (Magiati et al. 2017; Renno and Wood 2013). However, given the limited cognitive and communication abilities of people with rare genetic syndromes associated with ID, properly attributing symptoms and differentiating diagnoses (e.g., autism and anxiety) can be a complex process (Appleton et al. 2019).

One factor that may underlie the relationship between autism and anxiety in CdLS and FXS is intolerance of uncertainty (IU). IU is characterised by distress or difficulty functioning during uncertain or unpredictable situations, a strong preference for predictability, and cognitive or behavioural paralysis or inhibition (Berenbaum et al. 2008; Birrell et al. 2011; Freeston et al. 1994). IU has been identified as an important risk factor for the development and maintenance of anxiety in the general population (Carleton 2012) and has been implicated as a critical factor underpinning anxiety (Boelen and Reijntjes 2009; Counsell et al. 2017; Freeston et al. 1994; Norr et al. 2013; Ozsivadjian et al. 2021; Whiting et al. 2014). Additionally, studies have identified IU as a causal factor in the development and maintenance of anxiety in autistic people (Boulter et al. 2014; Cai et al. 2018; Hwang et al. 2020; Jenkinson et al. 2020; Maisel et al. 2016; Neil et al. 2016; Rodgers, Glod, et al. 2012; Rodgers and Ofield 2018; Wigham et al. 2015) and people with Williams syndrome (Uljarević et al. 2018). Furthermore, IU has also been identified as a mediating factor between anxiety and specific autistic characteristics such as sensory processing, social differences and social communication difficulties in both autistic people and people with Williams syndrome (Boulter et al. 2014; Hwang et al. 2020; MacLennan et al. 2021; Neil et al. 2016; South et al. 2021; Uljarević et al. 2018; Vasa et al. 2018; Wigham et al. 2015). Studies suggest that this relationship warrants investigation in CdLS and FXS. One study noted that IU was significantly associated with autistic characteristics for both groups (Groves et al. 2022). Furthermore, people with CdLS and FXS display a strong preference for routine and sameness (Moss et al. 2016; Moss et al. 2009; Richards et al. 2017; Roberts et al. 2016; Wolff et al. 2012), anxiety‐related behaviours that resemble components of IU (e.g., distress or difficulty functioning during uncertain or unpredictable situations; Rodgers et al. 2017). Understanding the nature of, and the factors underpinning, anxiety in CdLS and FXS is important for the development of tailored interventions to reduce anxiety in rare genetic syndromes associated with ID.

Growing evidence suggests that IU is an important risk factor for the development and maintenance of anxiety in autistic people, yet there is a paucity of research on the impact of IU on the autism–anxiety relationship in rare genetic syndromes such as CdLS and FXS. Given the association between autism and CdLS and FXS, it is plausible that a similar relationship may exist between autism, anxiety and IU in these groups. Understanding this relationship is essential to informing theoretical frameworks of anxiety in rare genetic syndromes and improving interventions. This is particularly important given that parents of autistic children have reported that their child's anxiety contributes to limitations in engagement in enjoyable activities, poorer parental wellbeing and negative health outcomes for their child (Tarver et al. 2021), outcomes that may also be relevant to people with CdLS and FXS. This study aimed to (1) compare autistic characteristics, anxiety symptom severity and levels of IU in people with CdLS and FXS, (2) determine if there was a relationship between autistic characteristics, anxiety symptom severity and levels of IU in CdLS and FXS, and (3) investigate whether IU mediated the relationship between autistic characteristics and anxiety symptom severity in CdLS and FXS.

## 1 Methods

### 1.1 Recruitment

Participants with CdLS and FXS were recruited from a larger ongoing study investigating anxiety in rare genetic syndromes. Parents/carers were contacted through an existing database held at the Cerebra Centre for Neurodevelopmental Disorders at the University of Birmingham compiled through recruitment via appropriate syndrome support groups (e.g., Cornelia de Lange Foundation UK and Ireland, and the Fragile X Society). Families that met the inclusion criteria were contacted and invited to participate in the present study.

### 1.2 Participants

Participants were 31 people with CdLS and 29 males with FXS and were included if they had a confirmed diagnosis of CdLS or FXS from a medical professional (GP, clinical geneticist or paediatrician) and were at least 4 years old. Females with FXS were excluded due to phenotypic sex differences, precluding between‐group comparisons. Participant demographics are displayed in Table 1. Participants were comparable for chronological age and adaptive behaviour ability, as determined by the Communication and Adaptive Behaviour Composite scores on the Vineland Adaptive Behaviour Scale, Second Edition (VABS‐II; Sparrow et al. 2005).

**TABLE 1:** Descriptive characteristics.

	CdLS (*n* = 31)	FXS (*n* = 29)	*U*	*p*
Median age, years	13.10	20.42	341.5	0.110
Range	3.75–53.50	6.67–46.92	—	—
Sex, *n* (% male)	18 (58.06%)	29 (100%)	—	—
Median VABS Communication Subscale	56	44	375	0.265
Range	20–74	21–87		
Median VABS Adaptive Behaviour Composite score	56	52	395	0.420
Range	22–75	20–73		

### 1.3 Measures

#### 1.3.1 Demographic Information

Parents/carers completed a demographics questionnaire confirming their child's age, sex, verbal ability, mobility status and details of diagnosis (e.g., name of genetic syndrome, genetic mechanism causing the syndrome, age of diagnosis, who provided diagnosis).

#### 1.3.2 Measures of Ability

The VABS‐II (Sparrow et al. 2005) is a parent/carer interview measure that assesses adaptive behaviour in four key domains: Daily Living, Communication, Socialisation, and Motor Skills. Though designed for neurotypical children (birth‐18 years), the VABS‐II has been widely used with individuals with ID. The VABS‐II Adaptive Behaviour Composite score, derived from domain‐specific scores, was used to provide an overall estimate of adaptive behaviour, with lower scores indicative of poorer adaptive functioning (e.g., 70 or below suggests poor adaptive functioning). Internal consistency for the Adaptive Behaviour Composite score was high in children and adults with ID (Cronbach's alpha = 0.99; De Bildt et al. 2005). Cronbach's alpha for the present study was excellent for the CdLS (α = 0.94) and FXS group (α = 0.93).

#### 1.3.3 Autistic Characteristics

The Social Responsiveness Scale, Second Edition (SRS‐2) (Constantino and Gruber 2012), a 65‐item rating scale, assessed autistic characteristics across five subscales: Social Awareness, Social Cognition, Social Communication, Social Motivation, and Restricted Interests and Repetitive Behaviour. Parents/carers completed the age‐appropriate form [i.e., preschool, school‐age or adult (relative/other report)]. The items across each form differ slightly to accommodate differences with age; however, each of the items is equivalent and targets the same behaviour (Bruni 2014). The SRS‐2 has good inter‐rater reliability (*r* = 0.75–0.91) and is not related to IQ (Constantino et al. 2003; Constantino et al. 2007). Although raw scores are typically used in research (Constantino and Gruber 2012), *T‐*scores were used to account for sex differences which may lead to overestimation of impairment in unaffected males (Hus et al. 2013) and ensure group comparability. *T*‐scores have been used in other studies exploring autistic characteristics across a wide age range (Bruni 2014) as well as in people with rare genetic syndromes (e.g., Channell 2020; Groves et al. 2022). Cronbach's alpha for the present study was excellent for the CdLS group (α = 0.93) and good for the FXS group (α = 0.87).

#### 1.3.4 Anxiety Symptom Severity

Anxiety symptom severity was measured using the Anxiety, Depression, and Mood Scale (ADAMS; Esbensen et al. 2003), a 28‐item informant report designed to screen for anxiety, depression and mood disorders among children and adults with ID across five subscales: Manic/Hyperactive Behaviour, Depressed Mood, Social Avoidance, General Anxiety, and Compulsive Behaviour. The General Anxiety subscale (ADAMS‐GA) was used as a measure of overall anxiety (Cordeiro et al. 2011). ADAMS‐GA subscale scores are not standardised and are calculated by summing the relevant items. Higher subscale scores indicate greater anxiety symptom severity. While there are no clinical cutoffs for the ADAMS, a recommended threshold of ≥ 10 has been suggested for identifying clinically significant generalised anxiety in adults with ID (Hermans et al. 2012). The ADAMS has strong reliability for each of the subscales (mean Cronbach's alpha = 0.80) and robust test–retest correlations at the scale and subscale level (mean subscale = 0.78). Cronbach's alpha for the present study was good for the CdLS group (α = 0.88) and acceptable for the FXS group (α = 0.71).

#### 1.3.5 Intolerance of Uncertainty

Levels of IU were assessed using the Intolerance of Uncertainty Scale—Parent Version (IUS‐P; Rodgers, Freeston, et al. 2012), a 12‐item informant questionnaire used to assess an individual's ability to cope with uncertainty in particular situations. The measure yields an unstandardised total score, for which higher scores indicate greater levels of IU. The scale has excellent internal consistency for neurotypical and autistic people (Boulter et al. 2014). Cronbach's alpha for the present study was excellent for the CdLS group (α = 0.92) and good for the FXS group (α = 0.83).

### 1.4 Procedures

Families received information sheets, consent forms and questionnaires at syndrome support group meetings or home visits as part of the larger study. Ethical approval was granted by NHS Coventry and Warwickshire Research Ethics Committee (REC reference 16/WM/0435) as part of the larger ongoing cross‐syndrome study.

### 1.5 Data Analysis

SRS‐2 *T*‐scores, ADAMS‐GA subscale scores, and IUS‐P total scores were examined for normality using stem‐and‐leaf plots and Shapiro–Wilk tests, confirming normal distribution. A post hoc power analysis was conducted (G*Power; Faul et al. 2007) with a sample size of 60 and a 3‐predictor variable equation with a medium effect size (*f* ^*2*^ = 0.15; Cohen 1977). Statistical power for this study was 0.12 for small effects, 0.68 for medium effects and > 0.97 for large effects, indicating sufficient power only for large effects. Therefore, results (especially nonsignificant) should be interpreted with caution.

## 2 Results

### 2.1 Group Differences in Autistic Characteristics, Anxiety and IU

To address the first aim, independent samples *t*‐tests were used to compare autistic characteristics, anxiety symptom severity and levels of IU in people with CdLS and FXS. No significant differences were found for autistic characteristics as measured by the SRS‐2 *T‐*scores (CdLS: M = 70.97, SD = 11.01; FXS: M = 74.00, SD = 7.93; *t*(54.55) = −1.23, *p* = 0.22), anxiety symptom severity as measured by the ADAMS‐GA subscale scores (CdLS: M = 8.04, SD = 4.98; FXS: M = 7.92, SD = 4.44; *t*(58) = 0.10, *p* = 0.92), or levels of IU as measured by the IUS‐P total score (CdLS: M = 31.65, SD = 13.27; FXS: M = 32.17, SD = 9.90; *t*(58) = −0.17, *p* = 0.86).

### 2.2 Relationship Between Autistic Characteristics, Anxiety and IU

To address the second aim to determine if there was a relationship between autistic characteristics, anxiety symptom severity and levels of IU in CdLS and FXS, a hierarchical multiple regression analysis was conducted to determine whether autistic characteristics, level of IU or syndrome group predicted anxiety symptom severity as measured by the ADAMS‐GA subscale scores. Multicollinearity was low between predictors (tolerance: 0.88–0.98; variance inflation factor: 1.03–1.14) and the assumption of independent errors was tenable (Durbin–Watson = 2.13). In the first step of the hierarchical multiple regression analysis, autistic characteristics significantly predicted anxiety symptom severity (*F*(1, 58) = 12.22, *p* < 0.001), explaining 16% of the variance in anxiety. In the second step of the analysis, levels of IU significantly predicted anxiety symptom severity (*F*(2, 57) = 33.16, *p* < 0.001), accounting for an additional 36% of the variance in anxiety after controlling for autistic characteristics (*R* ^2^ = 0.54, *FChange* (1, 57) = 44.87, *p* < 0.001). In the third step of the analysis, the model was statistically significant when syndrome group was entered (*F*(3, 56) = 22.03, *p* < 0.001); however, the total variance explained by the model as a whole remained at 52% (*R* ^2^ = 0.54, *FChange* (1, 56) = 0.43, *p =* 0.514). Full results are displayed in Table 2. Results indicate that while autistic characteristics, levels of IU and syndrome group significantly contribute to the variance in anxiety symptom severity, only autistic characteristics and levels of IU predicted anxiety symptom severity.

**TABLE 2:** Hierarchical linear models for ADAMS‐GA subscale scores.

	B	SE	*B*	*p*	*R*	*R* ^2^	Adj *R* ^2^
**Step 1**					**0.42**	**0.17**	**0.16**
Constant	−6.65	4.22		0.121			
SRS‐2 *T*‐scores	0.20	0.06	0.42	< 0.001			
**Step 2**					**0.73**	**0.53**	**0.52**
Constant	−7.07	3.19		0.030			
SRS‐2 *T*‐scores	0.09	0.05	0.19	0.048			
IUS‐P total scores	0.26	0.04	0.64	< 0.001			
**Step 3**					**0.74**	**0.54**	**0.52**
Constant	−4.89	4.61		0.294			
SRS‐2 *T*‐scores	0.10	0.05	0.20	0.041			
IUS‐P total scores	0.26	0.04	0.64	< 0.001			
Syndrome group	−0.56	0.85	−0.06	0.514			

### 2.3 IU as a Mediating Factor

To address the third aim, mediation analyses were used to investigate whether IU mediated the relationship between autistic characteristics and anxiety symptom severity in CdLS and FXS. Separate mediation analyses were conducted with the CdLS and FXS groups to evaluate syndrome‐specific relationships between autistic characteristics, anxiety and IU.

**FIGURE 1:**
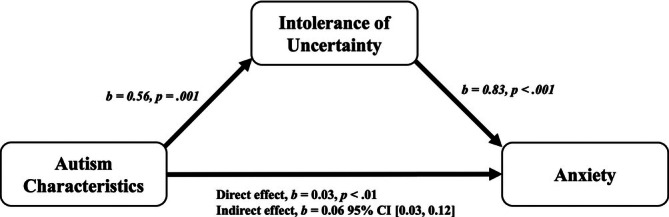
Mediation model between intolerance of uncertainty, autism characteristics and anxiety symptom severity in people with CdLS.

**FIGURE 2:**
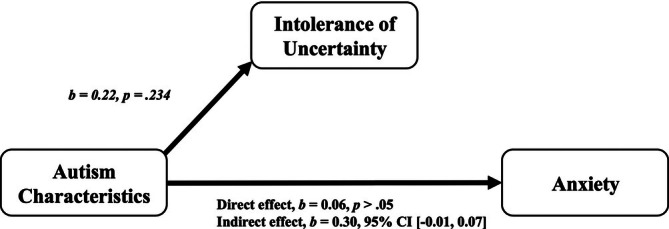
Mediation model between intolerance of uncertainty, autism characteristics and anxiety symptom severity in people with FXS.

Mediation analyses were conducted using a computational tool for mediation and moderation (SPSS macro PROCESS; Preacher and Hayes 2004). This method uses a bootstrapping procedure to obtain estimates and confidence intervals around the indirect effects. Significant relationships in the models are indicated by bootstrapped confidence intervals that do not overlap with zero. The direct and indirect effects of autistic characteristics on anxiety symptom severity with the mediator variable (IU) considered are outlined in Table 3. For the CdLS group, there was a significant indirect effect of autistic characteristics on anxiety through the mediating variable of IU [*b* = 0.14 (95% bootstrapped CI 0.03, 0.30)], indicating that IU fully mediated the relationship between autistic characteristics and anxiety. However, for the FXS group, the indirect effect of autistic characteristics on anxiety through IU was not significant [*b* = 0.05 (95% bootstrapped CI −0.07, 0.17)]. Results of the mediation analyses were consistent using Baron and Kenny's (1986) approach (see Table 4). Results indicate that IU fully mediated the relationship between autistic characteristics and anxiety in people with CdLS but did not in males in FXS. Visual representations of the mediation analyses are displayed in Figures 1 and 2 to illustrate the major findings.

**TABLE 3:** Direct and indirect effects of autistic characteristics on anxiety in CdLS and FXS.

		Direct effect			Indirect effect		
		*B*	SE	95% CI	*B*	SE	95% CI
CdLS	IU	0.08	0.06	−0.05 to 0.20	0.14**	0.07	0.03 to 0.30
FXS	IU	0.14	0.08	−0.02 to 0.31	0.05	0.06	−0.07 to 0.17

** *p* < 0.01.

**TABLE 4:** Summary of statistical analyses and significant regressions for each step of the causal steps model in CdLS and FXS groups.

				*F*	*df*	*R* ^2^	Beta	*p*
	Mediating model for ADAMS‐GA subscale scores	Step 1		8.48	1, 29	0.226	0.215	0.007
		Step 2		7.34	1, 29	0.202	0.542	0.011
CdLS		Step 3		39.17	1, 29	0.575	0.285	< 0.001
		Step 4	SRS‐2 *T*‐scores	20.78	2, 28	0.597	0.077	0.218
			IUS‐P total scores				0.256	< 0.001
FXS	Mediating model for ADAMS‐GA subscale scores	Step 1		3.66	1, 27	0.119	0.193	0.067
		Step 2		0.58	1, 27	0.021	0.181	0.453

## 3 Discussion

Given that people with rare genetic syndromes are likely to experience co‐occurring autism and anxiety, understanding the autism‐anxiety relationship is essential to informing interventions and theoretical frameworks for anxiety in rare genetic syndromes associated with ID. IU has been identified as a mechanism of anxiety in the general population (Boelen and Reijntjes 2009; Carleton 2012; Counsell et al. 2017; Freeston et al. 1994; Norr et al. 2013; Ozsivadjian et al. 2021; Whiting et al. 2014), autistic people (Boulter et al. 2014; Cai et al. 2018; Hwang et al. 2020; Jenkinson et al. 2020; Maisel et al. 2016; Neil et al. 2016; Rodgers, Glod, et al. 2012; Rodgers and Ofield 2018; Wigham et al. 2015), and people with Williams syndrome (Uljarević et al. 2018). Due to the heightened likelihood of experiencing co‐occurring autism and anxiety in people with rare genetic syndromes, this study investigated the relationships between autism, anxiety and IU in people with CdLS and FXS.

Results showed that people with CdLS and FXS did not significantly differ on autistic characteristics (indexed by SRS‐2 *T*‐scores), anxiety symptom severity (indexed by ADAMS‐GA subscale scores), or levels of IU (indexed by IUS‐P total scores). Autistic characteristics significantly accounted for the variance in anxiety symptom severity, an effect which was maintained after levels of IU and syndrome group were considered. Similarly, levels of IU accounted for a large part of the variance in anxiety symptom severity, an effect that was maintained after syndrome group was considered. Syndrome group also significantly contributed to the variance in anxiety symptom severity. Despite the initial similarities, mediation analyses revealed differences in the autism‐anxiety relationship between groups. For the CdLS group, IU fully mediated the relationship between autistic characteristics and generalised anxiety. This relationship was not observed in the FXS, with results indicating that IU is unlikely to mediate the relationship between autistic characteristics and generalised anxiety.

These findings suggest that the relationship between autistic characteristics, anxiety and IU in CdLS is comparable to the relationship observed in autistic people, whereas in FXS, this relationship is less clear. This is somewhat surprising given that, similar to CdLS, FXS is a rare genetic syndrome strongly associated with co‐occurring autism and anxiety. It is possible that sex differences could be influencing the results, as FXS is an X‐linked disorder, and this study only included male participants to reduce phenotypic sex differences. Although there are notable sex differences observed in people with FXS (Rinehart et al. 2011), both males and females with FXS experience high levels of co‐occurring anxiety (Bailey et al. 2008; Cordeiro et al. 2011). However, recent studies in autism indicate mixed results regarding the relationship between sex and anxiety, with some studies suggesting that sex was related to anxiety scores (with autistic females reporting more anxiety compared with autistic males; Bernardin et al. 2021; Murray et al. 2019; Uljarević et al. 2021) and others finding no relationship between sex and anxiety (Gadow et al. 2008; Kerns et al. 2014; Mayes et al. 2011; Pickard et al. 2020; Sáez‐Suanes et al. 2023). Given the mixed results in the autism literature, future research examining sex and gender differences in rare genetic syndromes such as FXS would be beneficial and would better capture within‐syndrome variability. In addition to sex and gender differences, genotypic differences may help to explain and better capture within‐syndrome variability given that both CdLS and FXS result from different genetic mutations. Future research should also aim to include more detailed genotypic information to capture within‐syndrome variability among rare genetic syndromes.

Another possibility is that the *profiles* of autistic characteristics and anxiety differed between CdLS and FXS. Although there were no significant differences between the CdLS and FXS groups in overall degree of autistic characteristics or anxiety symptom severity, it is possible that differences in the profiles of the subscale scores may have impacted the relationship between autistic characteristics, anxiety and IU. Previous studies support this assumption, highlighting the heterogeneity in the profile of autistic characteristics (Bozhilova et al. 2023; Moss and Howlin 2009) and anxiety (Crawford et al. 2017; Groves et al. 2022) in rare genetic syndromes associated with ID. Additionally, clearly identifying and distinguishing anxiety from autism may be complicated due to behavioural and diagnostic overlap (Edwards et al. 2022; van Steensel et al. 2011) as well as autism‐specific presentations of anxiety (Kerns et al. 2014). To account for these differences, future research should investigate the relationship between different aspects of the autism and anxiety profiles in CdLS and FXS to determine which factors may be influencing the autism‐anxiety relationship in these two groups.

While the present study examined IU, there may be other factors mediating the relationship between autistic characteristics and anxiety in people with FXS. For example, sensory sensitivity has been identified as a mediating factor between IU and anxiety in autistic people (Hwang et al. 2020; MacLennan et al. 2021; Neil et al. 2016; Wigham et al. 2015) and people with Williams syndrome (Uljarević et al. 2018). Given that hypersensitivity to sensory stimuli is also a characteristic of FXS, it is possible that a similar association may be present. Furthermore, Crawford et al. (2017) suggested that sensory hypersensitivity may contribute to the presence of anxiety disorders in people with FXS, particularly in highly stimulating environments. Similarly, social skills have also been identified as a mediating factor between IU and anxiety in autistic people and people with Williams syndrome (South et al. 2021). Reisinger and Roberts (2017) found that boys with FXS displayed significant impairments in social skills compared with neurotypical peers at the same age, which may be confounded by co‐occurring autism and anxiety. Additionally, there is evidence to suggest that physiological hyperarousal differentially predicts both autistic characteristics and anxiety in young children with FXS (Hogan et al. 2021). Although the exact mechanism remains unclear, it is possible that physiological hyperarousal underpins the autism‐anxiety relationship in people with FXS and may be worth exploring as a possible mediating factor between autistic characteristics and anxiety (Crawford 2023). Therefore, it is possible that other factors, such as sensory sensitivity, social skills and hyperarousal, may be influencing the relationship between autistic characteristics, anxiety and IU in FXS, but additional research is needed to delineate this relationship.

The present study has some limitations that are common in research into genetic syndromes associated with ID. The study relied on informant‐report measures. Boulter et al. (2014) used both parent‐report and child‐report questionnaires to assess anxiety and IU in autistic children without co‐occurring ID, finding strong correlations and good agreement between parent and child scores. However, it was unlikely that many of the participants in the present study would have been able to complete self‐report questionnaires or reflect on their own experiences of anxiety (Cordeiro et al. 2011). Additionally, participants in the present study varied in age, with the inclusion of both children and adults; therefore, presentations of anxiety may have differed due to developmental differences (Spence 1998). However, Mian et al. (2012) demonstrated that neurotypical children, as young as 2 years of age, showed similar clusters of anxiety symptoms (consistent with the DSM‐5 anxiety disorder diagnostic criteria) to adolescents, suggesting that anxiety presentation and differentiation remain relatively stable over time.

It is also worth considering limitations regarding the scores used to assess autistic characteristics, intolerance of uncertainty and anxiety. For example, SRS *T‐*scores were used to assess autistic characteristics in people with CdLS and males with FXS. Although SRS *T‐*scores have been used to explore autistic characteristics across a wide age range (Bruni 2014) as well as in people with rare genetic syndromes (e.g., Channell 2020; Groves et al. 2022), the use of *T‐*scores may limit potentially significant findings. This is particularly important for males with FXS as the use of *T*‐scores may reduce sensitivity given that many males with FXS score at or near the upper limit of the SRS, which may consequentially weaken any observed associations between autistic characteristics, intolerance of uncertainty and anxiety. Future research may benefit from exploring alternative scoring methods, such as raw scores, to enhance sensitivity to individual differences that may be present in different syndrome groups.

Another limitation is that the ADAMS has not been validated for autistic people. Moreover, there are few measures of anxiety validated for both children and adults with ID and autism. The ADAMS has been validated in participants five to 33 years old with FXS (Cordeiro et al. 2011) and was therefore considered a good measure to identify anxiety in CdLS and FXS. While this measure is not ideal, research into rare genetic syndromes often comes with compromises regarding measures, as it is unusual to find a measure that has been specifically validated in a particular syndrome. While alternative measures of anxiety, such as the Anxiety Scale for Children with Autism Spectrum Disorder (ASC‐ASD; Rodgers et al. 2016), have been used in people with CdLS and FXS to provide insight into autism‐specific anxiety (Groves et al. 2022), this measure has not been validated for use in adults or people with ID. Furthermore, the ASC‐ASD contains an uncertainty subscale, which may confound the predictor and outcome variable of the present study, making it difficult to accurately parse the relationship between autistic characteristics, anxiety and IU. Ideally, direct measures of anxiety, such as behavioural observations or physiological measures (i.e., heart rate), would have been included in the present study but were not feasible due to time and recruitment restraints.

The results of the present study suggest IU plays a key role in the presence of anxiety in CdLS, highlighting IU as a potential target for anxiety intervention in this population. Previous studies indicate that cognitive behavioural therapy (CBT) targeting IU effectively reduces generalised anxiety symptomatology in typical development (Dugas et al. 2010; Zemestani et al. 2021). Moreover, interventions such as ‘Coping with Uncertainty in Everyday Situations’ (CUES; Rodgers et al. 2017), developed for autistic children and later adapted for autistic adults (CUES‐A; Rodgers et al. 2018), have shown promise in effectively reducing IU in autistic people (Rodgers et al. 2023; Rodgers et al. 2017). The development of autism‐specific interventions targeting IU, such as the CUES and CUES‐A, is promising for people with CdLS. Further work is needed to validate the efficacy of these programmes in people with rare genetic syndromes (e.g., CdLS). It is also unclear whether these interventions, or similar interventions, would be appropriate for people with CdLS or FXS as there are currently limited accessible or appropriate intervention options for people with more moderate to profound ID (Vereenooghe et al. 2018). Future research should continue to focus on understanding the autism‐anxiety relationship and developing interventions that reduce anxiety and improve the quality of life for people with CdLS, FXS and other syndrome groups, particularly for those with more moderate to severe ID.

To conclude, the results from the present study suggest that the relationship between autistic characteristics, anxiety and levels of IU differs for people with CdLS and males with FXS. While the CdLS and FXS groups did not differ in autistic characteristics, anxiety symptom severity or levels of IU, levels of IU fully mediated the relationship between autism characteristics and anxiety symptom severity for the CdLS group, but not for the FXS group. These findings improve our understanding of the autism–anxiety relationship and can inform future research directions, as well as potential new interventions and theoretical frameworks, improving quality of life for individuals with rare genetic syndromes and their families.

## Ethics Statement

All procedures performed in the current study were in accordance with the ethical standards of the institutional research committee and with the principles of the Declaration of Helsinki and its later amendments. The study received approval from NHS Coventry and Warwickshire Research Ethics Committee (REC reference 16/WM/0435).

## Consent

Informed consent was obtained from all participants included in the study.

## Conflicts of Interest

The authors declare no conflicts of interest.

## Data Availability

The data that support the findings of this study are available from the corresponding author upon reasonable request. The data are not publicly available because of privacy or ethical restrictions.

## Endnotes

^1^
The terminology ‘autistic characteristics’ was used to describe overall characteristics and behaviours associated with autism. This terminology was used to avoid potentially ableist language and is in line with recommendations from Bottema‐Beutel et al. (2021) and autism charities, such as Autistica (Autistica n.d.).
